# 3-[(1-Benzyl-1*H*-1,2,3-triazol-5-yl)meth­yl]-6-bromo-2-phenyl-3*H*-imidazo[4,5-*b*]pyridine

**DOI:** 10.1107/S1600536811009123

**Published:** 2011-03-15

**Authors:** Younes Ouzidan, Youssef Kandri Rodi, Frank R. Fronczek, Ramaiyer Venkatraman, El Mokhtar Essassi, Lahcen El Ammari

**Affiliations:** a Laboratoire de Chimie Organique Appliquée, Université Sidi Mohamed Ben Abdallah, Faculté des Sciences et Techniques, Route d’Immouzzer, BP 2202 Fès, Morocco; b Department of Chemistry, Louisiana State University, Baton Rouge, LA 70803, USA; c Department of Chemistry and Biochemistry, Jackson State University, Jackson, MS 39217, USA; d INANOTECH (Institute of Nanomaterials and Nanotechnology), MAScIR, Avenue de l’Armée Royale, Rabat, Morocco; e Laboratoire de Chimie du Solide Appliquée, Faculté des Sciences, Université Mohammed V-Agdal, Avenue Ibn Battouta, BP 1014, Rabat, Morocco

## Abstract

There are two crystallographically independent mol­ecules in the asymmetric unit of the title compound, C_22_H_17_BrN_6_. The dihedral angles between the imidazo[4,5-*b*]pyridine mean plane and the phenyl rings are 20.4 (2) and 24.0 (2)° in the two mol­ecules. The orientation of triazoles compared to the imidazo[4,5-*b*]pyridine system is almost the same in both mol­ecules, with dihedral angles of 64.2 (2) and 65.1 (2)°. However, the main difference between the two mol­ecules lies in the arrangement of the phenyl groups compared to imidazo[4,5-*b*]pyridine in each mol­ecule. Indeed, in the first mol­ecule the dihedral angle between the plane of the phenyl ring and that of the imidazo[4,5-*b*]pyridine system is 67.7 (2)°, while in the second mol­ecule the plane of the phenyl ring is almost perpendicular to that of the imidazo[4,5-*b*]pyridine system with a dihedral angle of 86.0 (2)°.

## Related literature

For background literature on the medicinal properties of imidazo[4,5-*b*]pyridine and its derivatives, see: Jiyeon *et al.* (2010 ▶); Passannanti *et al.* (1998 ▶); Bavetsias *et al.* (2007 ▶); Tomczuk *et al.* (1991 ▶); Ouzidan, Obbade *et al.* (2010 ▶); Ouzidan, Rodi *et al.* (2010 ▶).
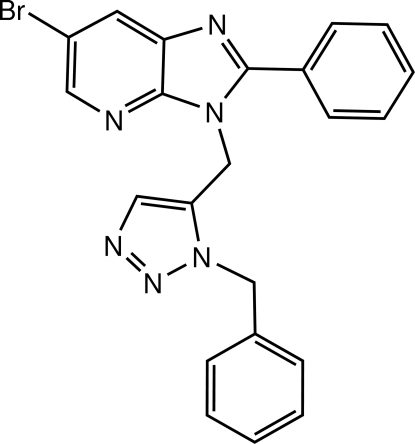


## Experimental

### 

#### Crystal data

C_22_H_17_BrN_6_
*M* *_r_* = 445.33
Monoclinic, 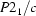

*a* = 41.122 (6) Å
*b* = 5.8358 (10) Å
*c* = 15.988 (3) Å
β = 93.922 (6)°
*V* = 3827.8 (11) Å^3^
*Z* = 8
Mo *K*α radiation
μ = 2.17 mm^−1^
*T* = 93 K
0.32 × 0.05 × 0.01 mm

#### Data collection

Nonius KappaCCD diffractometer
Absorption correction: multi-scan (*SCALEPACK*; Otwinowski & Minor, 1997 ▶) *T*_min_ = 0.878, *T*_max_ = 0.979
14153 measured reflections
7510 independent reflections
4016 reflections with *I* > 2σ(*I*)
*R*_int_ = 0.088

#### Refinement

*R*[*F* ^2^ > 2σ(*F* ^2^)] = 0.048
*wR*(*F* ^2^) = 0.109
*S* = 0.96
7510 reflections
524 parameters
H-atom parameters constrained
Δρ_max_ = 0.37 e Å^−3^
Δρ_min_ = −0.43 e Å^−3^

### 

Data collection: *COLLECT* (Nonius, 2000 ▶); cell refinement: *SCALEPACK* (Otwinowski & Minor, 1997 ▶); data reduction: *DENZO* (Otwinowski & Minor, 1997 ▶) and *SCALEPACK*; program(s) used to solve structure: *SIR97* (Altomare *et al.*, 1999 ▶); program(s) used to refine structure: *SHELXL97* (Sheldrick, 2008 ▶); molecular graphics: *ORTEPIII* (Burnett & Johnson, 1996 ▶), *ORTEP-3 for Windows* (Farrugia, 1997 ▶) and *PLATON* (Spek, 2009 ▶)’; software used to prepare material for publication: *SHELXL97*.

## Supplementary Material

Crystal structure: contains datablocks I, global. DOI: 10.1107/S1600536811009123/dn2663sup1.cif

Structure factors: contains datablocks I. DOI: 10.1107/S1600536811009123/dn2663Isup2.hkl

Additional supplementary materials:  crystallographic information; 3D view; checkCIF report

## Figures and Tables

**Fig. 1.**
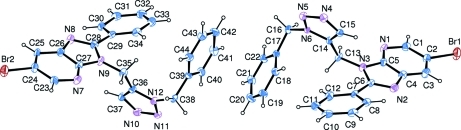
Plot of the asymmetric unit of the title compound with the atom-labelling scheme. Displacement ellipsoids are drawn at the 50% probability level. H atoms are represented as small circles.

**Fig. 2.**
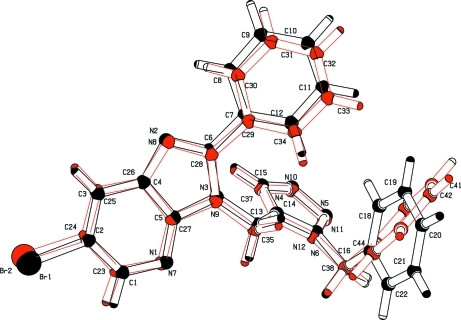
View showing the fitting of the two molecules building the asymmetric unit.

## supplementary crystallographic information

### Comment

This work is a result of an extensive program of scientific research on the synthesis and characterization of imidazo[4,5-*b*]pyridine and its derivatives started in the laboratory since two years (Ouzidan, Obbade *et al.* 2010; Ouzidan, Rodi *et al.* 2010). It is motivated by numerous applications of these compounds in medicinal chemistry (Passannanti *et al.*, (1998); Tomczuk *et al.*, (1991)). Furthermore, the imidazo[4,5-*b*]pyridine moiety is also an important heterocyclic nucleus which has been used extensively in medicinal chemistry. In fact, the heterocycles derived from these intermediates have been tested for their potential as anti-neuroinflammatory (Jiyeon *et al.*, (2010); Bavetsias *et al.*, (2007).

In this work we have synthesized 3-((3-benzyl-3*H*-1,2,3-triazol-4-yl)methyl)- 6-bromo-2-phenyl-3*H*-imidazo[4,5-*b*]pyridine by 1,3-dipolar cycloaddition reaction of benzyl azide with 6-bromo-2-phenyl-3-(prop-2-ynyl)-3*H*-imidazo [4,5-*b*]pyridine.

The plot of the two molecules building the asymmetric unit of the crystal structure of 3-((3-benzyl-3*H*-1,2,3-triazol-4-yl)methyl)-6-bromo-2- phenyl-3*H*-ιmidazo[4,5-*b*]pyridine is shown in Fig. 1. The two cycles forming the imidazo[4,5-*b*]pyridine are almost planar and form dihedral angles with the phenyl rings of 20.4 (2)° and 24.0 (2)°, in the first and in the second molecule respectively. Moreover, the dihedral angle between the phenyl ring and the imidazo[4,5-*b*]pyridine system is 67.7 (2) °, in the first while in the second molecule the phenyl is almost perpendicular to the imidazo[4,5-*b*]pyridine system with a dihedral angle of 86.0 (2) ° as shown in Fig.2 which represents the fitting of the two molecules.

### Experimental

To a solution of 6-bromo-2-phenyl-3-(prop-2-ynyl)-3*H*-imidazo[4,5-*b*]pyridine (0.2 g, 0.64 mmol) in ethanol (15 ml) was added benzyl azide (0.1 ml, 0.77 mmol). The mixture was stirred under reflux for 72 h. After completion of reaction (monitored by TLC), the solution was concentrated and the residue was purified by column chromatography on silica gel by using a mixture (hexane/ethyl acetate 2/1). Crystals were obtained when the solvent was allowed to evaporate.

### Refinement

H atoms were located in a difference map and treated as riding with C—H = 0.93 Å for all H atoms with *U*_iso_(H) = 1.2 *U*_eq_(aromatic, methine) and *U*_iso_(H) = 1.5 *U*_eq_(methyl).

The reflections 002, -102 and -202 were omitted because the difference between their calculated and observed intensities are very large. They are affected by the beamstop.

### Crystal data

**Table:**

C_22_H_17_BrN_6_	*F*(000) = 1808
*M**_r_* = 445.33	*D*_x_ = 1.545 Mg m^−^^3^
Monoclinic, *P*2_1_/*c*	Mo *K*α radiation, λ = 0.71073 Å
Hall symbol: -P 2ybc	Cell parameters from 8098 reflections
*a* = 41.122 (6) Å	θ = 2.5–26.0°
*b* = 5.8358 (10) Å	µ = 2.17 mm^−^^1^
*c* = 15.988 (3) Å	*T* = 93 K
β = 93.922 (6)°	Lath, colourless
*V* = 3827.8 (11) Å^3^	0.32 × 0.05 × 0.01 mm
*Z* = 8	

### Data collection

**Table:**

Nonius KappaCCD diffractometer	7510 independent reflections
Radiation source: fine-focus sealed tube	4016 reflections with *I* > 2σ(*I*)
graphite	*R*_int_ = 0.088
ω and φ scans	θ_max_ = 26.0°, θ_min_ = 2.6°
Absorption correction: multi-scan (*SCALEPACK*; Otwinowski & Minor, 1997)	*h* = −50→50
*T*_min_ = 0.878, *T*_max_ = 0.979	*k* = −7→7
14153 measured reflections	*l* = −19→19

### Refinement

**Table:**

Refinement on *F*^2^	Secondary atom site location: difference Fourier map
Least-squares matrix: full	Hydrogen site location: inferred from neighbouring sites
*R*[*F*^2^ > 2σ(*F*^2^)] = 0.048	H-atom parameters constrained
*wR*(*F*^2^) = 0.109	*w* = 1/[σ^2^(*F*_o_^2^) + (0.0383*P*)^2^] where *P* = (*F*_o_^2^ + 2*F*_c_^2^)/3
*S* = 0.96	(Δ/σ)_max_ = 0.002
7510 reflections	Δρ_max_ = 0.37 e Å^−^^3^
524 parameters	Δρ_min_ = −0.43 e Å^−^^3^
0 restraints	Extinction correction: *SHELXL97* (Sheldrick, 2008), Fc^*^=kFc[1+0.001xFc^2^λ^3^/sin(2θ)]^-1/4^
Primary atom site location: structure-invariant direct methods	Extinction coefficient: 0.00033 (9)

### Special details

**Table:**

Geometry. All s.u.'s (except the s.u. in the dihedral angle between two l.s. planes) are estimated using the full covariance matrix. The cell s.u.'s are taken into account individually in the estimation of s.u.'s in distances, angles and torsion angles; correlations between s.u.'s in cell parameters are only used when they are defined by crystal symmetry. An approximate (isotropic) treatment of cell s.u.'s is used for estimating s.u.'s involving l.s. planes.
Refinement. Refinement of *F*^2^ against ALL reflections. The weighted *R*-factor *wR* and goodness of fit *S* are based on *F*^2^, conventional *R*-factors *R* are based on *F*, with *F* set to zero for negative *F*^2^. The threshold expression of *F*^2^ > 2σ(*F*^2^) is used only for calculating *R*-factors(gt) *etc*. and is not relevant to the choice of reflections for refinement. *R*-factors based on *F*^2^ are statistically about twice as large as those based on *F*, and *R*- factors based on ALL data will be even larger.

### Fractional atomic coordinates and isotropic or equivalent isotropic displacement parameters (Å^2^)

**Table:**

	*x*	*y*	*z*	*U*_iso_*/*U*_eq_	
Br1	0.989447 (10)	−0.09151 (9)	0.32192 (3)	0.03656 (17)	
N1	0.90534 (7)	0.0612 (6)	0.4335 (2)	0.0193 (8)	
N2	0.94688 (8)	0.5152 (6)	0.5460 (2)	0.0230 (9)	
N3	0.89663 (7)	0.3576 (5)	0.5350 (2)	0.0187 (8)	
N4	0.81767 (8)	0.8153 (6)	0.4658 (2)	0.0207 (9)	
N5	0.79371 (7)	0.6850 (6)	0.4916 (2)	0.0194 (8)	
N6	0.80743 (7)	0.4880 (5)	0.5204 (2)	0.0149 (8)	
C1	0.92866 (9)	−0.0255 (7)	0.3886 (3)	0.0219 (11)	
H1	0.9233	−0.1522	0.3530	0.026*	
C2	0.96043 (9)	0.0590 (7)	0.3909 (3)	0.0252 (11)	
C3	0.97024 (10)	0.2447 (7)	0.4406 (3)	0.0248 (12)	
H3	0.9918	0.3045	0.4420	0.030*	
C4	0.94644 (10)	0.3375 (7)	0.4886 (3)	0.0208 (11)	
C5	0.91541 (9)	0.2381 (7)	0.4805 (3)	0.0179 (10)	
C6	0.91729 (9)	0.5220 (7)	0.5727 (3)	0.0193 (10)	
C7	0.90790 (9)	0.6912 (7)	0.6361 (3)	0.0177 (10)	
C8	0.92746 (9)	0.8867 (7)	0.6463 (3)	0.0231 (11)	
H8	0.9456	0.9056	0.6133	0.028*	
C9	0.92049 (10)	1.0533 (7)	0.7044 (3)	0.0260 (11)	
H9	0.9343	1.1832	0.7123	0.031*	
C10	0.89335 (10)	1.0306 (7)	0.7510 (3)	0.0259 (11)	
H10	0.8880	1.1478	0.7890	0.031*	
C11	0.87404 (10)	0.8355 (8)	0.7419 (3)	0.0280 (11)	
H11	0.8557	0.8181	0.7743	0.034*	
C12	0.88160 (10)	0.6658 (8)	0.6852 (3)	0.0269 (11)	
H12	0.8686	0.5312	0.6801	0.032*	
C13	0.86240 (8)	0.2979 (7)	0.5432 (3)	0.0194 (10)	
H13A	0.8591	0.2613	0.6025	0.023*	
H13B	0.8568	0.1599	0.5092	0.023*	
C14	0.84022 (9)	0.4920 (7)	0.5147 (2)	0.0167 (10)	
C15	0.84631 (9)	0.7005 (7)	0.4797 (3)	0.0179 (10)	
H15	0.8672	0.7559	0.4672	0.021*	
C16	0.78661 (9)	0.3022 (7)	0.5469 (3)	0.0178 (10)	
H16A	0.7914	0.1619	0.5151	0.021*	
H16B	0.7635	0.3435	0.5328	0.021*	
C17	0.79114 (9)	0.2506 (7)	0.6397 (3)	0.0176 (10)	
C18	0.80353 (8)	0.4096 (7)	0.6972 (2)	0.0191 (9)	
H18	0.8103	0.5550	0.6781	0.023*	
C19	0.80625 (9)	0.3609 (7)	0.7828 (3)	0.0224 (10)	
H19	0.8152	0.4709	0.8217	0.027*	
C20	0.79574 (9)	0.1489 (7)	0.8105 (3)	0.0261 (11)	
H20	0.7974	0.1133	0.8686	0.031*	
C21	0.78273 (9)	−0.0108 (7)	0.7529 (3)	0.0235 (10)	
H21	0.7753	−0.1546	0.7718	0.028*	
C22	0.78062 (9)	0.0389 (7)	0.6684 (2)	0.0199 (10)	
H22	0.7719	−0.0716	0.6294	0.024*	
Br2	0.504814 (10)	1.09139 (8)	1.17516 (3)	0.03284 (16)	
N7	0.59172 (7)	0.9600 (6)	1.0742 (2)	0.0224 (9)	
N8	0.55365 (8)	0.5067 (6)	0.9533 (2)	0.0220 (9)	
N9	0.60306 (7)	0.6754 (6)	0.9706 (2)	0.0197 (8)	
N10	0.68098 (8)	0.2184 (6)	1.0476 (2)	0.0225 (9)	
N11	0.70526 (7)	0.3468 (6)	1.0243 (2)	0.0201 (9)	
N12	0.69174 (7)	0.5464 (5)	0.9948 (2)	0.0170 (8)	
C23	0.56715 (9)	1.0389 (7)	1.1175 (3)	0.0241 (11)	
H23	0.5713	1.1634	1.1550	0.029*	
C24	0.53579 (9)	0.9465 (7)	1.1099 (3)	0.0224 (11)	
C25	0.52746 (9)	0.7637 (7)	1.0584 (3)	0.0249 (12)	
H25	0.5062	0.6992	1.0544	0.030*	
C26	0.55258 (9)	0.6800 (7)	1.0124 (3)	0.0209 (11)	
C27	0.58294 (9)	0.7867 (7)	1.0236 (3)	0.0193 (10)	
C28	0.58399 (9)	0.5082 (7)	0.9293 (3)	0.0191 (10)	
C29	0.59501 (9)	0.3528 (7)	0.8654 (3)	0.0194 (10)	
C30	0.57769 (9)	0.1458 (7)	0.8524 (3)	0.0215 (11)	
H30	0.5599	0.1129	0.8852	0.026*	
C31	0.58624 (10)	−0.0106 (7)	0.7924 (3)	0.0239 (11)	
H31	0.5742	−0.1485	0.7840	0.029*	
C32	0.61243 (10)	0.0343 (8)	0.7446 (3)	0.0282 (12)	
H32	0.6185	−0.0741	0.7042	0.034*	
C33	0.62961 (10)	0.2366 (7)	0.7558 (3)	0.0268 (12)	
H33	0.6473	0.2682	0.7225	0.032*	
C34	0.62105 (9)	0.3933 (7)	0.8154 (3)	0.0232 (10)	
H34	0.6331	0.5315	0.8225	0.028*	
C35	0.63732 (8)	0.7354 (6)	0.9684 (3)	0.0203 (10)	
H35A	0.6421	0.8705	1.0047	0.024*	
H35B	0.6419	0.7776	0.9104	0.024*	
C36	0.65910 (9)	0.5413 (7)	0.9975 (2)	0.0167 (10)	
C37	0.65276 (10)	0.3341 (7)	1.0321 (3)	0.0203 (10)	
H37	0.6318	0.2795	1.0435	0.024*	
C38	0.71226 (9)	0.7200 (6)	0.9582 (3)	0.0175 (10)	
H38A	0.7351	0.7008	0.9812	0.021*	
H38B	0.7048	0.8743	0.9741	0.021*	
C39	0.71105 (9)	0.7025 (7)	0.8641 (3)	0.0160 (10)	
C40	0.72190 (9)	0.5023 (7)	0.8270 (3)	0.0222 (10)	
H40	0.7306	0.3811	0.8612	0.027*	
C41	0.71999 (10)	0.4796 (7)	0.7407 (3)	0.0260 (11)	
H41	0.7272	0.3423	0.7159	0.031*	
C42	0.70759 (9)	0.6569 (7)	0.6903 (3)	0.0268 (11)	
H42	0.7064	0.6414	0.6310	0.032*	
C43	0.69698 (9)	0.8569 (7)	0.7266 (3)	0.0245 (11)	
H43	0.6887	0.9792	0.6923	0.029*	
C44	0.69857 (9)	0.8778 (7)	0.8139 (3)	0.0209 (10)	
H44	0.6910	1.0139	0.8388	0.025*	

### Atomic displacement parameters (Å^2^)

**Table:**

	*U*^11^	*U*^22^	*U*^33^	*U*^12^	*U*^13^	*U*^23^
Br1	0.0235 (3)	0.0446 (3)	0.0428 (4)	−0.0063 (2)	0.0115 (2)	−0.0184 (3)
N1	0.0119 (18)	0.019 (2)	0.027 (2)	−0.0002 (16)	0.0013 (15)	−0.0017 (18)
N2	0.020 (2)	0.023 (2)	0.026 (2)	−0.0010 (16)	−0.0012 (16)	−0.0046 (18)
N3	0.0155 (18)	0.018 (2)	0.023 (2)	0.0021 (15)	0.0017 (15)	−0.0005 (17)
N4	0.022 (2)	0.0176 (19)	0.022 (2)	−0.0036 (17)	0.0022 (16)	−0.0014 (17)
N5	0.0189 (19)	0.016 (2)	0.023 (2)	0.0011 (16)	0.0019 (16)	0.0037 (17)
N6	0.0142 (18)	0.0159 (19)	0.015 (2)	0.0010 (15)	0.0028 (14)	0.0025 (16)
C1	0.022 (2)	0.019 (3)	0.024 (3)	−0.0044 (19)	−0.0015 (19)	0.000 (2)
C2	0.019 (2)	0.032 (3)	0.025 (3)	−0.001 (2)	0.0027 (19)	0.004 (2)
C3	0.019 (2)	0.020 (3)	0.034 (3)	−0.005 (2)	0.002 (2)	−0.002 (2)
C4	0.020 (2)	0.016 (3)	0.026 (3)	−0.0012 (19)	0.000 (2)	0.002 (2)
C5	0.015 (2)	0.018 (3)	0.020 (3)	0.0010 (19)	0.0035 (19)	0.002 (2)
C6	0.014 (2)	0.019 (2)	0.024 (3)	−0.0004 (19)	−0.0035 (19)	0.001 (2)
C7	0.012 (2)	0.019 (2)	0.022 (3)	0.0027 (19)	−0.0028 (18)	0.002 (2)
C8	0.020 (2)	0.023 (3)	0.026 (3)	0.002 (2)	−0.0028 (19)	0.002 (2)
C9	0.026 (3)	0.024 (3)	0.028 (3)	0.001 (2)	−0.007 (2)	0.003 (2)
C10	0.031 (3)	0.019 (3)	0.027 (3)	0.011 (2)	−0.007 (2)	−0.002 (2)
C11	0.025 (3)	0.034 (3)	0.025 (3)	0.008 (2)	−0.001 (2)	−0.001 (2)
C12	0.020 (2)	0.027 (3)	0.033 (3)	−0.003 (2)	−0.001 (2)	0.001 (2)
C13	0.011 (2)	0.020 (2)	0.028 (3)	−0.0005 (18)	0.0003 (18)	0.002 (2)
C14	0.014 (2)	0.020 (2)	0.016 (2)	−0.0026 (18)	0.0027 (17)	0.000 (2)
C15	0.016 (2)	0.020 (2)	0.018 (3)	−0.0009 (19)	0.0008 (18)	0.002 (2)
C16	0.015 (2)	0.017 (2)	0.022 (3)	−0.0040 (18)	0.0037 (18)	0.001 (2)
C17	0.014 (2)	0.019 (3)	0.020 (3)	0.0029 (19)	0.0027 (18)	0.005 (2)
C18	0.020 (2)	0.017 (2)	0.021 (2)	0.003 (2)	0.0032 (17)	0.003 (2)
C19	0.025 (2)	0.023 (3)	0.019 (3)	0.002 (2)	0.0018 (18)	−0.003 (2)
C20	0.031 (3)	0.029 (3)	0.019 (3)	0.006 (2)	0.005 (2)	0.005 (2)
C21	0.026 (2)	0.020 (2)	0.026 (3)	0.003 (2)	0.0075 (19)	0.004 (2)
C22	0.021 (2)	0.021 (3)	0.018 (3)	0.0045 (19)	0.0022 (18)	−0.002 (2)
Br2	0.0237 (3)	0.0376 (3)	0.0383 (3)	−0.0037 (2)	0.0099 (2)	−0.0134 (3)
N7	0.0176 (19)	0.020 (2)	0.030 (2)	−0.0009 (16)	0.0006 (16)	−0.0009 (18)
N8	0.0173 (19)	0.021 (2)	0.028 (2)	−0.0014 (16)	0.0045 (16)	−0.0017 (18)
N9	0.0118 (18)	0.0175 (19)	0.030 (2)	−0.0006 (15)	0.0024 (16)	−0.0015 (18)
N10	0.022 (2)	0.024 (2)	0.022 (2)	−0.0012 (17)	−0.0017 (16)	−0.0006 (17)
N11	0.0177 (19)	0.024 (2)	0.019 (2)	0.0032 (16)	0.0003 (15)	0.0009 (17)
N12	0.0152 (18)	0.015 (2)	0.021 (2)	−0.0028 (15)	0.0001 (14)	−0.0006 (16)
C23	0.025 (2)	0.026 (3)	0.022 (3)	−0.002 (2)	0.0024 (19)	−0.004 (2)
C24	0.019 (2)	0.021 (3)	0.028 (3)	−0.003 (2)	0.0066 (19)	−0.001 (2)
C25	0.013 (2)	0.035 (3)	0.028 (3)	−0.006 (2)	0.0061 (19)	−0.005 (2)
C26	0.018 (2)	0.022 (3)	0.023 (3)	−0.0026 (19)	0.0015 (19)	0.002 (2)
C27	0.015 (2)	0.017 (2)	0.026 (3)	−0.0008 (19)	0.0001 (19)	0.001 (2)
C28	0.019 (2)	0.016 (2)	0.022 (3)	−0.0010 (19)	0.0018 (19)	0.002 (2)
C29	0.015 (2)	0.019 (3)	0.024 (3)	0.0018 (19)	−0.0043 (19)	−0.001 (2)
C30	0.016 (2)	0.025 (3)	0.023 (3)	0.005 (2)	0.0013 (18)	0.005 (2)
C31	0.026 (3)	0.020 (3)	0.025 (3)	0.000 (2)	−0.005 (2)	−0.001 (2)
C32	0.027 (3)	0.031 (3)	0.025 (3)	0.009 (2)	−0.003 (2)	−0.005 (2)
C33	0.022 (2)	0.031 (3)	0.028 (3)	0.007 (2)	0.002 (2)	0.002 (2)
C34	0.022 (2)	0.021 (3)	0.027 (3)	0.000 (2)	−0.0028 (19)	0.001 (2)
C35	0.018 (2)	0.013 (2)	0.030 (3)	−0.0042 (19)	0.0022 (19)	−0.001 (2)
C36	0.015 (2)	0.016 (3)	0.020 (3)	−0.0010 (18)	0.0005 (17)	−0.003 (2)
C37	0.015 (2)	0.024 (3)	0.022 (3)	−0.0022 (19)	0.0019 (18)	−0.001 (2)
C38	0.011 (2)	0.017 (2)	0.024 (3)	−0.0059 (18)	−0.0010 (18)	−0.001 (2)
C39	0.016 (2)	0.017 (2)	0.015 (3)	−0.0036 (19)	0.0006 (18)	−0.001 (2)
C40	0.023 (2)	0.023 (2)	0.021 (3)	0.0011 (19)	0.0050 (19)	0.001 (2)
C41	0.030 (3)	0.017 (2)	0.032 (3)	−0.001 (2)	0.009 (2)	−0.003 (2)
C42	0.029 (3)	0.032 (3)	0.019 (3)	−0.005 (2)	−0.001 (2)	−0.002 (2)
C43	0.023 (2)	0.018 (3)	0.033 (3)	0.0011 (19)	0.000 (2)	0.004 (2)
C44	0.022 (2)	0.018 (2)	0.022 (3)	−0.003 (2)	−0.0002 (18)	−0.003 (2)

### Geometric parameters (Å, °)

**Table:**

Br1—C2	1.895 (4)	Br2—C24	1.899 (4)
N1—C5	1.326 (5)	N7—C27	1.330 (5)
N1—C1	1.337 (5)	N7—C23	1.345 (5)
N2—C6	1.318 (5)	N8—C28	1.330 (5)
N2—C4	1.384 (5)	N8—C26	1.387 (5)
N3—C5	1.390 (5)	N9—C27	1.386 (5)
N3—C6	1.392 (5)	N9—C28	1.390 (5)
N3—C13	1.465 (4)	N9—C35	1.454 (4)
N4—N5	1.332 (4)	N10—N11	1.322 (4)
N4—C15	1.360 (4)	N10—C37	1.351 (5)
N5—N6	1.348 (4)	N11—N12	1.361 (4)
N6—C14	1.358 (4)	N12—C36	1.346 (4)
N6—C16	1.462 (4)	N12—C38	1.466 (4)
C1—C2	1.395 (5)	C23—C24	1.395 (5)
C1—H1	0.9500	C23—H23	0.9500
C2—C3	1.388 (5)	C24—C25	1.377 (5)
C3—C4	1.393 (5)	C25—C26	1.397 (5)
C3—H3	0.9500	C25—H25	0.9500
C4—C5	1.400 (5)	C26—C27	1.396 (5)
C6—C7	1.484 (6)	C28—C29	1.461 (5)
C7—C12	1.388 (6)	C29—C34	1.400 (5)
C7—C8	1.399 (5)	C29—C30	1.411 (5)
C8—C9	1.388 (5)	C30—C31	1.387 (5)
C8—H8	0.9500	C30—H30	0.9500
C9—C10	1.390 (6)	C31—C32	1.387 (6)
C9—H9	0.9500	C31—H31	0.9500
C10—C11	1.390 (6)	C32—C33	1.381 (6)
C10—H10	0.9500	C32—H32	0.9500
C11—C12	1.392 (6)	C33—C34	1.384 (6)
C11—H11	0.9500	C33—H33	0.9500
C12—H12	0.9500	C34—H34	0.9500
C13—C14	1.505 (5)	C35—C36	1.499 (5)
C13—H13A	0.9900	C35—H35A	0.9900
C13—H13B	0.9900	C35—H35B	0.9900
C14—C15	1.369 (5)	C36—C37	1.362 (5)
C15—H15	0.9500	C37—H37	0.9500
C16—C17	1.513 (5)	C38—C39	1.505 (5)
C16—H16A	0.9900	C38—H38A	0.9900
C16—H16B	0.9900	C38—H38B	0.9900
C17—C18	1.379 (5)	C39—C44	1.377 (5)
C17—C22	1.396 (5)	C39—C40	1.397 (5)
C18—C19	1.395 (5)	C40—C41	1.382 (5)
C18—H18	0.9500	C40—H40	0.9500
C19—C20	1.393 (5)	C41—C42	1.387 (5)
C19—H19	0.9500	C41—H41	0.9500
C20—C21	1.392 (5)	C42—C43	1.387 (6)
C20—H20	0.9500	C42—H42	0.9500
C21—C22	1.378 (5)	C43—C44	1.399 (6)
C21—H21	0.9500	C43—H43	0.9500
C22—H22	0.9500	C44—H44	0.9500
			
C5—N1—C1	113.1 (3)	C27—N7—C23	113.0 (3)
C6—N2—C4	105.5 (3)	C28—N8—C26	105.4 (3)
C5—N3—C6	105.6 (3)	C27—N9—C28	106.2 (3)
C5—N3—C13	121.0 (3)	C27—N9—C35	121.4 (3)
C6—N3—C13	133.4 (3)	C28—N9—C35	132.3 (3)
N5—N4—C15	108.6 (3)	N11—N10—C37	108.7 (3)
N4—N5—N6	107.0 (3)	N10—N11—N12	106.5 (3)
N5—N6—C14	110.9 (3)	C36—N12—N11	110.8 (3)
N5—N6—C16	119.5 (3)	C36—N12—C38	129.1 (3)
C14—N6—C16	129.5 (3)	N11—N12—C38	119.8 (3)
N1—C1—C2	123.9 (4)	N7—C23—C24	123.0 (4)
N1—C1—H1	118.1	N7—C23—H23	118.5
C2—C1—H1	118.1	C24—C23—H23	118.5
C3—C2—C1	121.7 (4)	C25—C24—C23	122.8 (4)
C3—C2—Br1	121.7 (3)	C25—C24—Br2	121.7 (3)
C1—C2—Br1	116.5 (3)	C23—C24—Br2	115.5 (3)
C2—C3—C4	115.6 (4)	C24—C25—C26	115.0 (4)
C2—C3—H3	122.2	C24—C25—H25	122.5
C4—C3—H3	122.2	C26—C25—H25	122.5
N2—C4—C3	132.7 (4)	N8—C26—C27	110.1 (4)
N2—C4—C5	110.0 (4)	N8—C26—C25	132.3 (4)
C3—C4—C5	117.3 (4)	C27—C26—C25	117.6 (4)
N1—C5—N3	125.4 (4)	N7—C27—N9	125.4 (4)
N1—C5—C4	128.5 (4)	N7—C27—C26	128.4 (4)
N3—C5—C4	106.1 (4)	N9—C27—C26	106.2 (4)
N2—C6—N3	112.9 (4)	N8—C28—N9	112.1 (4)
N2—C6—C7	122.0 (4)	N8—C28—C29	122.7 (4)
N3—C6—C7	125.1 (4)	N9—C28—C29	125.2 (4)
C12—C7—C8	119.0 (4)	C34—C29—C30	117.3 (4)
C12—C7—C6	124.4 (4)	C34—C29—C28	125.5 (4)
C8—C7—C6	116.6 (4)	C30—C29—C28	117.2 (4)
C9—C8—C7	120.4 (4)	C31—C30—C29	121.1 (4)
C9—C8—H8	119.8	C31—C30—H30	119.5
C7—C8—H8	119.8	C29—C30—H30	119.5
C8—C9—C10	120.1 (4)	C30—C31—C32	120.0 (4)
C8—C9—H9	119.9	C30—C31—H31	120.0
C10—C9—H9	119.9	C32—C31—H31	120.0
C11—C10—C9	119.7 (4)	C33—C32—C31	120.0 (4)
C11—C10—H10	120.1	C33—C32—H32	120.0
C9—C10—H10	120.1	C31—C32—H32	120.0
C10—C11—C12	120.0 (4)	C32—C33—C34	120.1 (4)
C10—C11—H11	120.0	C32—C33—H33	119.9
C12—C11—H11	120.0	C34—C33—H33	119.9
C7—C12—C11	120.7 (4)	C33—C34—C29	121.5 (4)
C7—C12—H12	119.7	C33—C34—H34	119.2
C11—C12—H12	119.7	C29—C34—H34	119.2
N3—C13—C14	111.1 (3)	N9—C35—C36	111.7 (3)
N3—C13—H13A	109.4	N9—C35—H35A	109.3
C14—C13—H13A	109.4	C36—C35—H35A	109.3
N3—C13—H13B	109.4	N9—C35—H35B	109.3
C14—C13—H13B	109.4	C36—C35—H35B	109.3
H13A—C13—H13B	108.0	H35A—C35—H35B	107.9
N6—C14—C15	104.7 (3)	N12—C36—C37	104.6 (4)
N6—C14—C13	123.4 (4)	N12—C36—C35	123.3 (4)
C15—C14—C13	132.0 (4)	C37—C36—C35	132.1 (4)
N4—C15—C14	108.9 (4)	N10—C37—C36	109.4 (4)
N4—C15—H15	125.5	N10—C37—H37	125.3
C14—C15—H15	125.5	C36—C37—H37	125.3
N6—C16—C17	113.3 (3)	N12—C38—C39	111.8 (3)
N6—C16—H16A	108.9	N12—C38—H38A	109.3
C17—C16—H16A	108.9	C39—C38—H38A	109.3
N6—C16—H16B	108.9	N12—C38—H38B	109.3
C17—C16—H16B	108.9	C39—C38—H38B	109.3
H16A—C16—H16B	107.7	H38A—C38—H38B	107.9
C18—C17—C22	119.0 (4)	C44—C39—C40	119.4 (4)
C18—C17—C16	122.3 (4)	C44—C39—C38	121.1 (4)
C22—C17—C16	118.5 (4)	C40—C39—C38	119.5 (4)
C17—C18—C19	121.2 (4)	C41—C40—C39	120.4 (4)
C17—C18—H18	119.4	C41—C40—H40	119.8
C19—C18—H18	119.4	C39—C40—H40	119.8
C20—C19—C18	119.1 (4)	C40—C41—C42	120.1 (4)
C20—C19—H19	120.4	C40—C41—H41	120.0
C18—C19—H19	120.4	C42—C41—H41	120.0
C21—C20—C19	119.9 (4)	C43—C42—C41	119.9 (4)
C21—C20—H20	120.1	C43—C42—H42	120.1
C19—C20—H20	120.1	C41—C42—H42	120.1
C22—C21—C20	120.2 (4)	C42—C43—C44	119.8 (4)
C22—C21—H21	119.9	C42—C43—H43	120.1
C20—C21—H21	119.9	C44—C43—H43	120.1
C21—C22—C17	120.5 (4)	C39—C44—C43	120.4 (4)
C21—C22—H22	119.7	C39—C44—H44	119.8
C17—C22—H22	119.7	C43—C44—H44	119.8
			
C15—N4—N5—N6	−0.9 (4)	C37—N10—N11—N12	0.7 (4)
N4—N5—N6—C14	1.1 (4)	N10—N11—N12—C36	−1.5 (4)
N4—N5—N6—C16	−175.0 (3)	N10—N11—N12—C38	−175.6 (3)
C5—N1—C1—C2	0.4 (6)	C27—N7—C23—C24	0.3 (6)
N1—C1—C2—C3	−0.5 (7)	N7—C23—C24—C25	1.1 (7)
N1—C1—C2—Br1	179.9 (3)	N7—C23—C24—Br2	−178.4 (3)
C1—C2—C3—C4	0.9 (6)	C23—C24—C25—C26	−1.4 (6)
Br1—C2—C3—C4	−179.5 (3)	Br2—C24—C25—C26	178.0 (3)
C6—N2—C4—C3	−178.7 (5)	C28—N8—C26—C27	0.0 (5)
C6—N2—C4—C5	0.7 (5)	C28—N8—C26—C25	178.5 (5)
C2—C3—C4—N2	178.3 (4)	C24—C25—C26—N8	−177.9 (4)
C2—C3—C4—C5	−1.1 (6)	C24—C25—C26—C27	0.5 (6)
C1—N1—C5—N3	−178.3 (4)	C23—N7—C27—N9	178.7 (4)
C1—N1—C5—C4	−0.8 (6)	C23—N7—C27—C26	−1.3 (6)
C6—N3—C5—N1	178.0 (4)	C28—N9—C27—N7	−179.6 (4)
C13—N3—C5—N1	−1.7 (6)	C35—N9—C27—N7	2.7 (6)
C6—N3—C5—C4	0.0 (4)	C28—N9—C27—C26	0.5 (5)
C13—N3—C5—C4	−179.7 (3)	C35—N9—C27—C26	−177.2 (3)
N2—C4—C5—N1	−178.3 (4)	N8—C26—C27—N7	179.7 (4)
C3—C4—C5—N1	1.2 (7)	C25—C26—C27—N7	1.0 (7)
N2—C4—C5—N3	−0.4 (5)	N8—C26—C27—N9	−0.3 (5)
C3—C4—C5—N3	179.1 (4)	C25—C26—C27—N9	−179.0 (4)
C4—N2—C6—N3	−0.7 (5)	C26—N8—C28—N9	0.3 (5)
C4—N2—C6—C7	179.7 (4)	C26—N8—C28—C29	−178.5 (4)
C5—N3—C6—N2	0.5 (5)	C27—N9—C28—N8	−0.5 (5)
C13—N3—C6—N2	−179.9 (4)	C35—N9—C28—N8	176.9 (4)
C5—N3—C6—C7	−180.0 (4)	C27—N9—C28—C29	178.3 (4)
C13—N3—C6—C7	−0.4 (7)	C35—N9—C28—C29	−4.4 (7)
N2—C6—C7—C12	−159.2 (4)	N8—C28—C29—C34	155.2 (4)
N3—C6—C7—C12	21.3 (6)	N9—C28—C29—C34	−23.5 (7)
N2—C6—C7—C8	20.2 (6)	N8—C28—C29—C30	−24.1 (6)
N3—C6—C7—C8	−159.3 (4)	N9—C28—C29—C30	157.3 (4)
C12—C7—C8—C9	−0.4 (6)	C34—C29—C30—C31	0.0 (6)
C6—C7—C8—C9	−179.8 (4)	C28—C29—C30—C31	179.3 (4)
C7—C8—C9—C10	−2.1 (6)	C29—C30—C31—C32	0.7 (6)
C8—C9—C10—C11	2.8 (6)	C30—C31—C32—C33	−1.1 (6)
C9—C10—C11—C12	−1.1 (6)	C31—C32—C33—C34	0.9 (6)
C8—C7—C12—C11	2.2 (6)	C32—C33—C34—C29	−0.2 (6)
C6—C7—C12—C11	−178.5 (4)	C30—C29—C34—C33	−0.3 (6)
C10—C11—C12—C7	−1.5 (6)	C28—C29—C34—C33	−179.5 (4)
C5—N3—C13—C14	−116.3 (4)	C27—N9—C35—C36	115.4 (4)
C6—N3—C13—C14	64.2 (6)	C28—N9—C35—C36	−61.6 (6)
N5—N6—C14—C15	−0.8 (4)	N11—N12—C36—C37	1.7 (4)
C16—N6—C14—C15	174.8 (4)	C38—N12—C36—C37	175.1 (4)
N5—N6—C14—C13	179.0 (4)	N11—N12—C36—C35	−179.4 (4)
C16—N6—C14—C13	−5.4 (6)	C38—N12—C36—C35	−6.1 (6)
N3—C13—C14—N6	−175.8 (4)	N9—C35—C36—N12	174.2 (4)
N3—C13—C14—C15	3.9 (6)	N9—C35—C36—C37	−7.2 (7)
N5—N4—C15—C14	0.4 (5)	N11—N10—C37—C36	0.4 (5)
N6—C14—C15—N4	0.2 (4)	N12—C36—C37—N10	−1.3 (5)
C13—C14—C15—N4	−179.6 (4)	C35—C36—C37—N10	179.9 (4)
N5—N6—C16—C17	−114.4 (4)	C36—N12—C38—C39	−76.5 (5)
C14—N6—C16—C17	70.3 (5)	N11—N12—C38—C39	96.3 (4)
N6—C16—C17—C18	22.8 (5)	N12—C38—C39—C44	116.3 (4)
N6—C16—C17—C22	−161.0 (3)	N12—C38—C39—C40	−61.8 (5)
C22—C17—C18—C19	1.2 (6)	C44—C39—C40—C41	−0.3 (6)
C16—C17—C18—C19	177.4 (4)	C38—C39—C40—C41	177.8 (3)
C17—C18—C19—C20	−1.1 (6)	C39—C40—C41—C42	0.7 (6)
C18—C19—C20—C21	0.0 (6)	C40—C41—C42—C43	−0.3 (6)
C19—C20—C21—C22	0.9 (6)	C41—C42—C43—C44	−0.6 (6)
C20—C21—C22—C17	−0.7 (6)	C40—C39—C44—C43	−0.5 (6)
C18—C17—C22—C21	−0.3 (6)	C38—C39—C44—C43	−178.6 (3)
C16—C17—C22—C21	−176.7 (4)	C42—C43—C44—C39	1.0 (6)
